# Tumor-Produced Versican V1 Enhances hCAP18/LL-37 Expression in Macrophages through Activation of TLR2 and Vitamin D3 Signaling to Promote Ovarian Cancer Progression *In Vitro*

**DOI:** 10.1371/journal.pone.0056616

**Published:** 2013-02-12

**Authors:** Dong Li, Xuan Wang, Jun-Lu Wu, Wen-Qiang Quan, Li Ma, Fan Yang, Kai-Yin Wu, Hai-Ying Wan

**Affiliations:** 1 Department of Clinical Laboratory, Tongji Hospital of Tongji University, Shanghai, China; 2 Department of Pharmacy, Putuo People's Hospital, Shanghai, China; 3 Institute of Pathology, Charité University Hospital, Berlin, Germany; Cedars-Sinai Medical Center, United States of America

## Abstract

Tumor-associated macrophages have been shown to promote tumor growth. They may have an obligatory function in angiogenesis, invasion, and metastasis through release of inflammatory mediators. Their presence in ovarian cancer has been correlated with poor prognosis in these patients. The human cationic antimicrobial protein-18 (hCAP18)/LL-37 was originally identified as an effector molecule of the innate immune system. It is released by innate immune cells, such as macrophages, to combat microorganisms. Previous studies have characterized the hCAP18/LL-37 as a growth factor that has been shown to promote ovarian tumor progression. However, the role hCAP18/LL-37 has in macrophage-promoted ovarian tumor development and how its expression is controlled in this context remains poorly understood. Here, we demonstrate in co-culture experiments of macrophages and ovarian cancer cells a significant increase in the *in vitro* proliferation and invasiveness of the tumor cells is observed. These enhanced growth and invasion properties correlated with hCAP18/LL-37 induction. HCAP18/LL-37 expression was diminished by addition of two neutralizing antibodies, TLR2 or TLR6, as well as Cyp27B1 or VDR inhibitors. Furthermore, either the TLR2 or TLR6 antibody reduced vitamin D3 signaling and tumor cell progression *in vitro*. Addition of Cyp27B1 or VDR inhibitors abrogated TLR2/6 activation-induced expression of hCAP18/LL-37 in macrophages. Knockdown of tumor-produced versican V1 by RNAi in these tumor cells led to a decreased induction of hCAP18/LL-37 in macrophages. Versican V1 knockdown also inhibited TLR2 and vitamin D3 signaling, as well as growth and invasiveness of these tumor cells in the *in vitro* co-culture. In summary, we have found that versican V1 enhances hCAP18/LL-37 expression in macrophages through activation of TLR2 and subsequent vitamin D-dependent mechanisms which promote ovarian tumor progression *in vitro*.

## Introduction

A tumor microenvironment plays a critical role in tumor initiation and promotion. This unique microenvironment contains innate immune cells, lymphocytes and connective tissue as well as malignant cells [1]. Innate immune cells (also known as myeloid cells), include macrophages, release cytokines and chemokines as well as influence tumorigenesis [1]–[3]. Tumor-associated macrophages (TAMs) are an important component of inflammatory infiltrates in tumors. TAMs are derived from circulating monocytic precursors by chemoattractants, secreted by both tumor and stromal cells [4], [5]. Clinical studies continue to demonstrate a correlation between a high populations of TAMs a poor prognosis in ovarian, breast, prostate, lung, and cervical cancers [6]–[8]. There is a growing body of evidence that several pro-inflammatory factors produced by macrophages, promote tumor growth, angiogenesis, invasion, and metastases [1], [5], [9]. However, the actual role a number of key pro-inflammatory molecules have in tumor promotion and their mechanisms of expression and function remains poorly understood.

The human cationic antimicrobial protein-18 (hCAP18)/LL-37 is the only known cathelicidin peptide in humans [10]. HCAP18/LL-37 is constitutively produced in numerous cell types including macrophages, neutrophils, skin epithelial cells of the skin, gastrointestinal tract and urinary tract, by the epididymis and by respiratory epithelial cells [11], [12]. The HCAP18 gene consists of 3 domains: the N-terminal signal peptide region, the highly conserved cathelin-like domain and the C-terminal region termed the LL-37 peptide [13]. The LL-37 peptide is maintained in its pro-peptide form until cleavage by protease 3 just prior to secretion [14], [15]. The LL-37 peptide serves various functions in different immune reactions, including immune modulation, inflammatory reaction, cell proliferation, angiogenesis, and antiapoptotic activity [16]. LL-37 has been established as a contributor to tumorigenesis and tumor progression [16], [17]. Studies have shown in ovarian cancers LL-37 contributes to cell proliferation, invasion, and cancer progression through direct stimulation of tumor cells, initiation of angiogenesis and recruitment of immune cells [14], [18], [19]. Treatment with a synthetic, biologically active LL-37 peptide or transgenic expression LL-37 significantly increases lung tumor cell proliferation. This research suggests that LL-37 acts as growth factor for human lung cancer [20]. Additionally, LL-37 also is considered to increase proliferation and metastasis in breast tumors and malignant melanomas [21], [22]. Taken together these findings support the hypothesis that LL-37 functions a growth factor in transformed cells.

A variety of stimuli, including pro-inflammatory molecules, growth factors, nutrients, bacterial products, and especially inflammation and injury up-regulate expression of hCAP18/LL-37 [16]. In humans 1,25-dihydroxyvitamin D3 (1,25D3) upregulates expression of hCAP18/LL-37 via the vitamin D receptor (VDR) in monocytes, macrophages, kerationocytes in the epidermis [23], [24]. Previous research that focused on intracellular *Mycobacterium tuberculosis* demonstrated TLR2 activation in human macrophages up-regulated expression of VDR and Cyp27B1 genes. This cascade of events increases the production of 1,25D3, which in turn leads to the induction of hCAP18/LL-37 [24]. Recent studies of the tumor microenvironment have demonstrated Lewis lung carcinoma (LLC) cells produced factors, such as versican, are necessary for lung tumor growth and metastasis. Moreover this process is dependent on TLR2-mediated myeloid cell activation [25], resulting in NF-κB activation of inflammatory factors TNFα, IL-6 production [26], [27].

The aim of this study is to investigate the regulation mechanisms of hCAP18/LL-37 in the tumor microenvironment. Here we report the versican V1 derived from tumor cells enhances hCAP18/LL-37 expression in macrophages through the activation of TLR2 and subsequent vitamin D-dependent mechanisms. Moreover it is this chain of cellular signaling events that promotes ovarian tumor cell proliferation and invasion. These results propose novel mechanism for hCAP18/LL-37 regulation in the tumor microenvironment. In addition they provide insights into critical factors involved in the cancer progression.

## Materials and Methods

### Cell lines and reagents

The human ovarian cancer cell lines OV-90 and SKOV3 cells were obtained from the American Type Culture Collection, and other human ovarian cancer cell lines HO-8910, 3AO cells were purchased from Shanghai Institute of Cell Biology, Chinese Academy of Science. These cells were cultured in Dulbeccos's modified Eagles medium (DMEM) (Hyclone laboratories. Inc, South, Utah, USA) supplemented with 10% fetal calf serum (FCS) (Invitrogen, Grand Island, NY, USA), 100 U/mL penicillin, and 100 U/mL streptomycin (Hyclone laboratories. Inc). Cell cultures were performed at 37°C in humidified air with 5% CO_2_. FCS was replaced with 10% complement-inactivated human serum (HS) (obtained from the blood bank of the Tongji Hospital of Tongji University. Institutional approval from the local research ethical committees (Internal Review and the Ethics Boards of the Tongji Hospital, Tongji University) was obtained prior to conducting this study) 24 hours before experiment. Neutralizing antibody anti-hCAP18/LL-37 (2 µg/ml, Clone # mAb 3D11, Hycult biotech,Netherland), anti-TLR2 (10 µg/ml, Clone # mAb 383936, R&D Systems, Minneapolis, MN, USA), anti-TLR6 (10 µg/ml, Clone # mAb C5C8, Invivogen, San Diego, CA, USA) and Cyp27B1 inhibitor itraconazole (10^−7^ M, Sigma Aldrich, St. Louis, MO) or VDR antagonist ZK159222 (10^−7^ M, a gift from Schering AG, Berlin, Germany) were added as indicated 2 hours before coculture or other stimulation. TLR2/6 ligand Pam2CSK4 was obtained from Invivogen. 25D3 (the 25-hydroxyvitamin D3, the 1,25D3 precursor) was purchased (BioMol, Plymouth Meeting, PA, USA) and resuspended in ethanol at 10^−2^ M in amber tubes and stored at −80°C in small aliquots.

### Generation of human peripheral blood monocyte-derived macrophages

Institutional approval from the local research ethical committees (Internal Review and the Ethics Boards of the Tongji Hospital, Tongji University) was obtained prior to conducting the study. Human peripheral blood monocyte-derived macrophages were generated as previously described [28]. Briefly, human peripheral blood mononuclear cells (PBMC) from healthy blood donors from the blood bank of the Tongji Hospital of Tongji University were isolated from buffy coats by Ficoll-Paque PLUS (GE Healthcare, Uppsala, Sweden) density centrifugation. PBMC were allowed to adhere to culture flasks for 1 h at 37°C in DMEM supplemented with 1% human serum, after which the nonadherent cells were removed by vigorous washing with PBS. Adherent cells were cultured in 20 ml DMEM (10% FCS) supplemented with 50 ng/ml macrophage colony stimulating factor (M-CSF) (eBioscience, San Diego, CA, USA) for 7 days to allow differentiation to macrophages.

### Coculturing ovarian cancer cells and macrophages

For coculture studies with cancer cells and macrophages, cancer cells were seeded into the bottom of multi-well cell culture plates and macrophages were placed in transwell inserts (0.4 µm, Corning Incorporated, Corning, NY, USA) with a membrane permeable for liquids but not for cells. Cells were incubated overnight in DMEM supplemented with 10% human serum. The transwells were inserted into the well of multi-well culture plate and cultured for indicated time.

### Cell number count

Monocyte-derived macrophages (1×10^4^ cells) and cancer cells (1×10^4^ cells) were seeded in 24-well trans-well or cell culture plates and incubated overnight. Cells were cocultured for 4 days and then transwell inserts (contain macrophages) were removed, Cancer cells were harvested by trypsinization and counted with a hemocytometer (Beckman Coulter, Fullerton, CA, USA).

### BrdU ELISA cell proliferation assay

Ovarian cancer cell proliferation was determined by using the commercially available Cell Proliferation ELISA, BrdU (colorimetric) Kit (Roche, Mannheim, Germany). After 4 days coculture with macrophages, transwell inserts (contain macrophages) were removed, and supernatants of tumor cells were aspirated and 400 µl/well growth media containing 10 µM BrdU was added. Cells were incubated for additional 2 h at 37°C. Labelling medium was removed by tapping off and the cells were fixed by adding 200 µl FixDenat. FixDenat solution was thoroughly removed by tapping. 400 µl/well anti-BrdU-POD working solution was added and cells were incubated for 90 min at RT. The antibody conjugate was removed by flicking off and wells were washed 3 times with PBS. After that, 400 µl/well of a substrate solution were added and the solution was incubated at RT for 5–30 min. 200 µl 3N H_2_SO_4_ was added to each well and the plates were incubated for 1 min on the shaker. The absorbance was measured using ELISA reader at 450 nm.

### Invasion assay

Invasion assay was performed using a BD BioCoat Matrigel Invasion Chamber (BD Biosciences,Bedford, MA, U.S.A.) with an 8 µm pore size PET membrane, uniformly coated with BD Matrigel Matrix as described [18], [29]. Serum-starved cancer cells were added to the upper chamber at a density of 1×10^4^ cells per well. 1×10^4^ macrophages were placed into the lower chamber. Chamber was filled with DMEM plus 10% HS. At the indicated time, the adherent cells on the bottom of the membrane were removed and pelleted by centrifugation. The cell pellet was resolved in 100 µl of PBS and spun down on the slides. After air-drying, the cytospins were stained with 5 µl DAPI (Vector Laboratories, Burlingame, CA, USA). Migrated cells per high power field were determined by fluorescence microscopy.

### SKOV3 or macrophage conditioned medium

Conditioned medium was collected from SKOV3 cells or human macrophages derived from peripheral blood monocytes incubated in serum-free DMEM for 24 h, and filtered through a 0.2 µm filter. SKOV3 conditioned medium (SKOV3-CM) samples were added to human primary macrophages for 24 h, after which various genes expression were assayed. Macrophage conditioned medium (M-CM) were added to human ovarian cancer cell lines for 24 h, and cell supernatants were measured by ELISA for versican V1.

### Cell transduction

Log growth SKOV3 cells were washed 1 time with PBS and resuspended at 2×10^7^ cells/ml in 1 ml of Gene Pulser electroporation buffer reagent (Bio-Rad, Hercules, CA, USA), mixed with 20 µg of versican V1 RNAi plasmids or mock RNAi plasmids (both from OriGene Technologies, Inc. Rockville, MD, USA). Electroporations were performed using a Gene-Pulser (Bio-Rad) at 280 V and 960 µF in 0.4 cm cuvette (Bio-Rad). The samples were transferred to culture flasks containing complete DMEM medium with10% FCS in 25 cm^2^ and incubated at 37°C in 5% CO_2_. 48 hours later, the growth medium was changed and puromycin (Invitrogen) was added at a concentration of 5 µg/ml. The culture medium was changed every 4 days using fresh growth medium (containing 5 µg/ml puromycin). After 4 weeks, positive polyclonal populations (pools) were identified based on western blot analysis for versican V1 expression. Individual positive clones were eventually isolated by limiting dilution analysis in 96-well plates.

### Western blotting

Western blot analysis was performed as described earlier [30]. Briefly, 30 µg total protein extracts were loaded on 10% SDS-polyacrylamide gels, subjected to electrophoresis, and blotted onto Hybond-C Extra membranes (Amersham Bioscience, Buckinghamshire, United Kingdom). The cell supernatants were first concentrated to 1/10 of their original volume by vacuum centrifugation and then subjected to 4–12% NU/PAGE gradient gels (Invitrogen). The primary antibodies included: rabbit anti-versican V1 (1∶1000; Abcam, Cambridge, UK) and rabbit anti-hCAP18/LL-37 (1∶500; Santa Cruz Biotechnology, Santa Cruz, CA, USA), mouse anti-β-actin (1∶10000; Sigma Aldrich, Steinheim, Germany). HRP-conjugated goat anti-rabbit (1∶5000; Santa Cruz Biotechnology) or rabbit anti-mouse (1∶1000; Dako, Glostrup, Denmark) were used as secondary antibody.

### RNA isolation and real-time PCR

Cell total RNAs were prepared with RNeasy plus mini kit (Qiagen, Santa Clarita, CA, USA) according to manufacturer's recommendation. Real time PCR reaction mixtures have been described previously [28]. Briefly, cDNA was synthesized by reverse transcription reaction using the First Strand cDNA synthesis kit (Invitrogen). Real-time PCR was performed using the QPCR SYBR Green Mix (Bio-Rad) on an AB 7300 Real time PCR system machine (AB Applied Biosystems, Singapore). The following PCR primers were used: β-actin, 5'-AGCCTCGCCTTTGCCGA-3' and 5'-CTGGTGCCTGGGGCG-3'; hCAP18/LL-37, 5'-TGGGCCTGGTGATGCCT-3' and 5'-CGATGTTCCTTCGACAGGAAGC-3'; VDR, 5'-AAGGACAACCGACGC CACT-3' and 5'-ACACACCTGTAGCCGTACTA-3'; Cyp27B1, 5'-ACCCGACAC GGAGACCTTC-3' and 5'-CACAGGTGCGACAACTGGTA-3'; Cpy24, 5'-CGCAG CGGCTGGAGAT-3' and 5'-ATGGCGTTTCTTCCGATGCC-3'; TLR1, 5'-AACCC ATTCCGCAGTACTCCA-3' and 5'-AAGGCCACGTTTGCTCTTTTC-3'; TLR2, 5'-CAATGATGCTGCCATTCTCAT-3' and 5'-ATTATCTTCCGCAGCTTGC A-3'; TLR3, 5'-ACAACTTTAGCACGGCTCTGGA-3' and 5'-ACCTCAACTGGGATC TCGTCA-3'; TLR4, 5'-AGTTTCCTGCAATGGATCAAGG-3' and 5'-CTGCTT ATCTGAAGGTGTTGCAC-3'; TLR6, CCCATTCCACAGAACAGCAT-3' and 5'-A TAAGTCCGCTGCGTCATGA-3'; CD14, 5'-TGTGAGCTGGACGATGAAGAT-3' and 5'-CAGACACACACTGGAAGGCTT-3'. Specificity of RT-PCR was controlled by ‘no reverse transcription’ controls and melting curve analysis. Quantitative PCR results were obtained using the ΔΔCT (cycle threshold) method. Data were normalized to β-actin levels in each sample.

### ELISA assay for versican

Samples of cell culture supernatants were analyzed by human versican ELISA (USCN life science, INC. Houston, TX, USA) according to the manufacturer's instructions. The detection limit of the assay was 0.107 ng/mL.

### Statistical analysis

Values are displayed as mean plus or minus SEM. Comparisons between groups were analyzed by the t test (two-sided). Results were considered statistically significant for P values less than 0.05.

## Results

### The expression of hCAP18/LL-37 in macrophages promote proliferation and invasiveness of ovarian cancer cells

**Figure 1:**
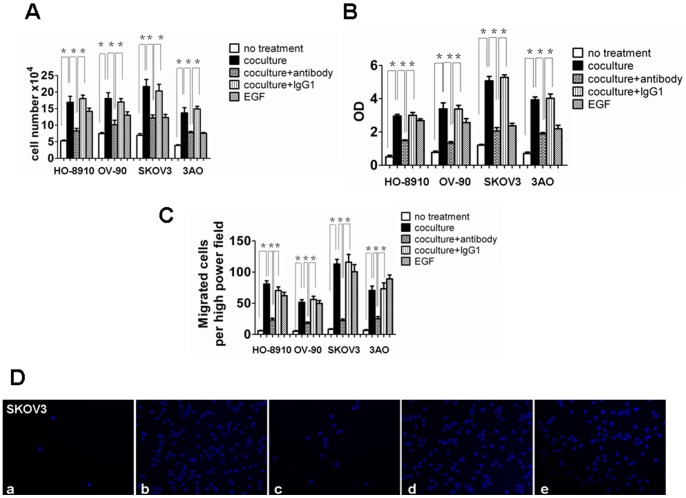
Cell proliferation and invasion is influenced by hCAP18/LL-37 in ovarian cancer cell lines. For co-culture experiments of ovarian cancer cells with macrophages, human peripheral blood monocyte-derived macrophages (1×10^4^ cells) were placed in transwell inserts and ovarian cancer cells (1×10^4^ cells) were seeded on the bottom of 24-well plates. Cells were preincubated with 2 µg/ml anti-hCAP18/LL-37 or an IgG1 isotype control Ab (2 µg/ml) for 2 h. The cells were co-cultured in DMEM (10%HS) for 4 days. 100 ng/ml EGF (Sigma, Steinheim, Germany) was used as control. (A) The proliferation of ovarian cancer cells was measured by cell number count. (B) Cell proliferation was measured by ELISA (BrdU labeling) analysis. (C) Invasion assay. Invasion assays was performed using a BD BioCoat Matrigel Invasion Chamber with an 8 µm pore size PET membrane, uniformly coated with BD Matrigel Matrix. Results are means ± SEM, significant difference, n = 3,*p<0.05. (D) Matrigel invasion assay of SKOV3. DAPI staining of migrated tumor cells. Representative sections are shown (a) SKOV3 no treatment. (b) SKOV3+macrophages. (c) SKOV3+macrophages+neutralizing anti-hCAP18/LL-37(2 µg/ml). (d) SKOV3+macrophages+IgG1 isotype control Ab (2 µg/ml). (e) SKOV3+EGF (100 ng/ml).

TAMs replaced with peripheral blood monocyte-derived macrophages for *in vitro* co-culture experiment had been reported by various groups. These reports show that peripheral blood monocyte-derived macrophages have equivalent function to TAMs [27], [31]. To determine the effect of macrophages on ovarian cancer cells proliferation, transwell inserts were used as a co-culture model. Human peripheral blood monocyte-derived macrophages were placed in transwell inserts and various ovarian cancer cell lines were seeded on the bottom of 24-well plates. Co-culture cells were grown for 4 days co-culture in DMEM containing 10% HS or treatment with EGF, as a positive control. The growth of HO-8910, OV-90, SKOV3, and 3AO cells were significantly increased when co-cultured with human macrophages derived from peripheral blood monocytes (Fig. 1A). To prevent hCAP18/LL-37 function monocyte cells were pretreated with a hCAP18/LL-37 neutralizing antibody before co-culture with ovarian cancer cells. In co-culture experiments where monocytes were pretreated with the hCAP18/LL-37 neutralizing antibody a significant inhibition of HO-8910, OV-90, SKOV3, and 3AO cells growth was observed (Fig. 1A). No cell growth inhibition was observed in the control IgG1 antibody co-culture experiments (Fig. 1A). To assess macrophage-induced ovarian cancer cells growth of BrdU incorporation into newly synthesized DNA strands measured using anti-BrdU antibodies in an ELISA assay. Consistent the observed increase in cell proliferation, DNA synthesis in HO-8910, OV-90, SKOV3, and 3AO cells was remarkably increased when cells were co-cultured with macrophages (Fig. 1B). As expected, addition of the hCAP18/LL-37 neutralizing antibody reduced the macrophage's effect on ovarian cancer cell proliferation (Fig. 1B). Next, ovarian cancer cells invasion was investigated. These cells' ability to invade Matrigel-coated inserts was also significantly enhanced by macrophages (Fig. 1C, D). After co-culture media was treated with the hCAP/LL-37 neutralizing antibody, it tumor cell invasion significantly attenuated (Fig. 1C, D). These results suggest a direct role for hCAP/LL-37 in promoting tumor cell invasion. An IgG control antibody did not interfere with ovarian cancer cell invasion (Fig. 1C, D). Taken together, these data suggest that hCAP18/LL-37 is required for macrophage-induced proliferation and invasion in ovarian cancer cells.

### Tumor cells trigger activation of vitamin D3 and TLR2 signaling and up regulation of hCAP18/LL-37 in macrophages

**Figure 2:**
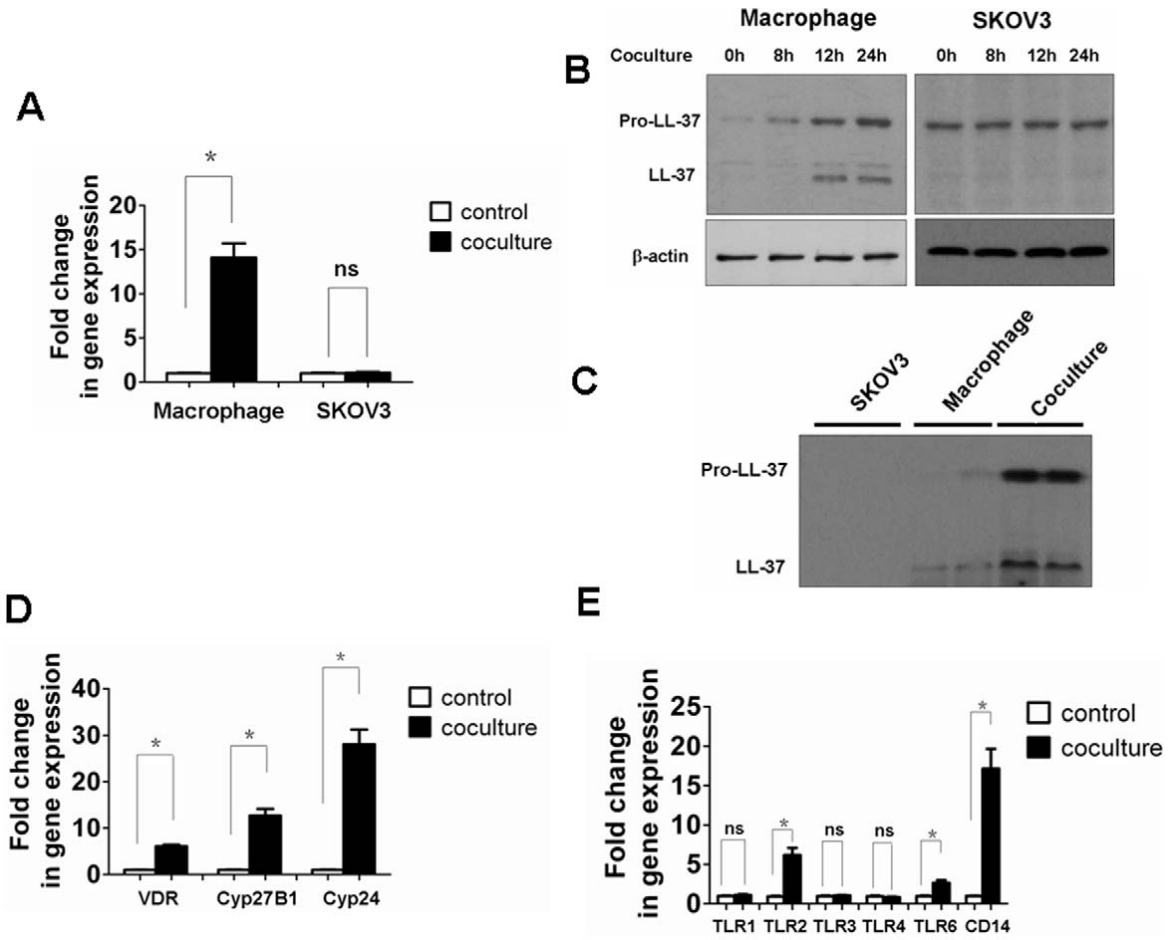
Tumor cells induce expression of hCAP18/LL-37, and activations of vitamin D3 and TLR2 signaling in macrophages. Human peripheral blood monocyte-derived macrophages and SKOV3 cells were coincubated as described in Figure 1. Total RNAs and proteins of SKOV3 cells and macrophages were isolated 24 h after co-culture. (A) The expression of hCAP18/LL-37 mRNA was measured by real-time PCR. (B) The expression of hCAP18/LL-37 protein was analyzed by western blot. β-actin served as loading control. (C) Western blot of cell supernatants from SKOV3 cells, macrophages, and co-culture at 24 h. (D,E) Induction of VDR, Cyp24, Cyp27B1, TLRs and CD14 mRNA was measured in macrophages by real-time PCR. Results are mean fold change ± SEM, significant difference, n = 3,*p<0.05; ns, not significant.

To investigate the expression pattern of hCAP18/LL-37, human macrophages were co-cultured with SKOV3 cells. The induction of hCAP18/LL-37 mRNA (Fig. 2A) and protein (full-length hCAP18/LL-37 and cleaved LL-37, Fig. 2B) levels increased in macrophages. Conversely, there were no significant differences in hCAP18/LL-37 mRNA or pro-peptide levels observed in SKOV3 cells (Fig. 2A, B). No, mature LL-37 was not detected in SKOV3 cells (Fig. 2B). These results indicated that macrophages and not SKOV3 cells contribute to the release of the LL-37 peptide. To assess the level of hCAP18/LL-37 and cleaved LL-37 in the cell medium, we performed western blotting on cell supernatants after 24 h of co-culture. A significant increase in the levels of the precursor and cleaved peptide were observed in comparison to macrophages or SKOV3 cells (Fig. 2C).

Given the VDR has been reported to mediate the expression of hCAP18/LL-37 [23], [24] we investigate if the expression of VDR and related genes are induced during co-culture. Indeed, an increase in VDR mRNA levels was observed when macrophages were co-cultured with SKOV3 cells (Fig. 2D). Moreover, the 1,25 D3 catabolic enzyme Cyp24 (also known as Cyp24A1) was induced in macrophages when they were co-cultured with tumor cells. Cyp27B1, which catalyzes the conversion of inactive provitamin D3 hormone (25D3) into the bioactive form (1,25D3) [24], mRNA levels were also up-regulated in the co-culture model (Fig. 2D). It has been reported that TLR2 activation leads to Vitamin D-dependent induction of hCAP18/LL-37 in macrophages [32]. TLR2, 6 and CD14 mRNA levels all showed the expected increase in co-cultured macrophages. The expression levels of TLR1, TLR3, and TLR4 were not changed in these macrophage cells (Fig. 2E).

### Ovarian cancer cell-mediated induction of hCAP18/LL-37, VDR, Cyp24, and Cyp27B1 in macrophages is dependent on TLR2 and TLR6

**Figure 3:**
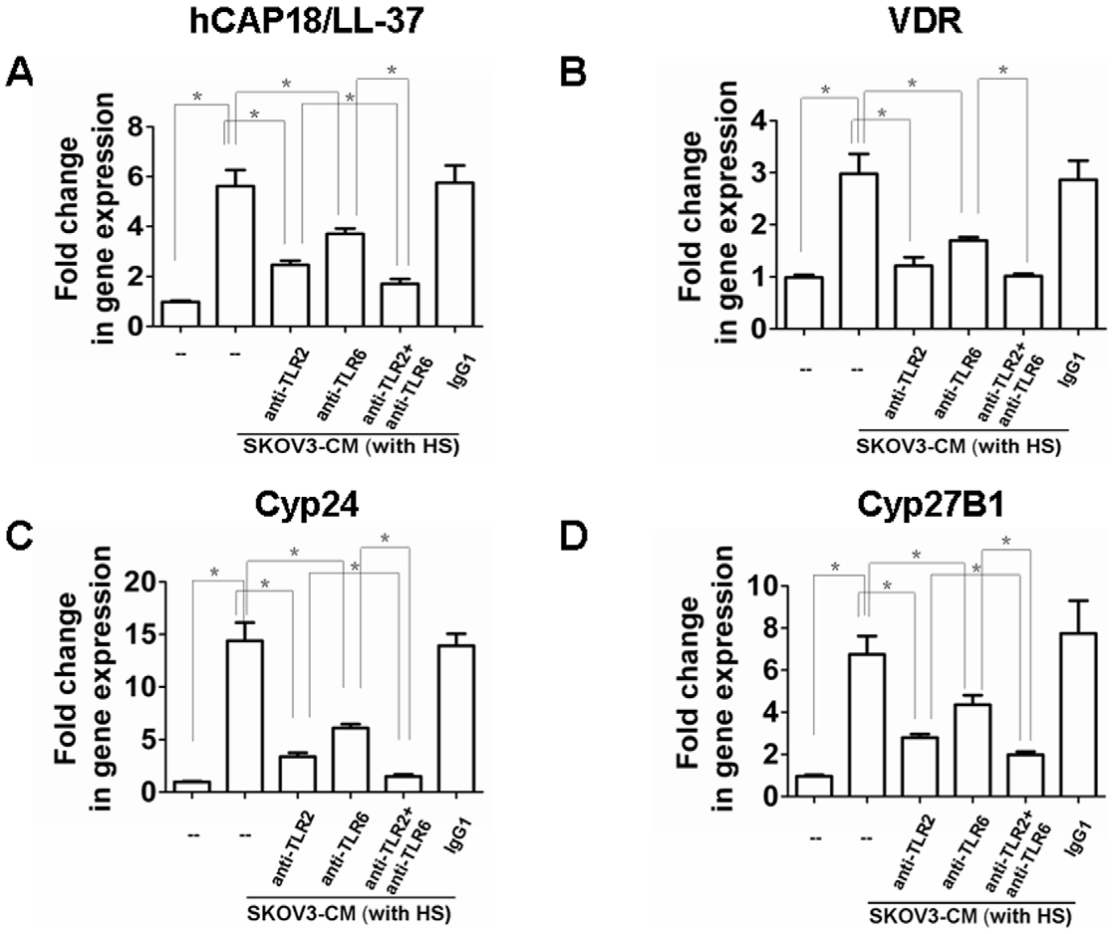
Responsiveness of macrophages to tumor cells is TLR2/6 dependent. Human macrophages were preincubated with 10 µg/ml anti-TLR2, 10 µg/ml anti-TLR6 or an IgG1 isotype control antibody (10 µg/ml) for 2 h. Then macrophages were culture with DMEM (10%HS) or SKOV3-CM (10%HS) for 24 h, and total RNAs were extracted for real-time PCR assay. (A) hCAP18/LL-37 mRNA. (B) VDR mRNA. (C) Cyp24 mRNA. (D) Cyp27B1 mRNA. Mean fold change ± SEM, n = 3, *p<0.05.

**Figure 4:**
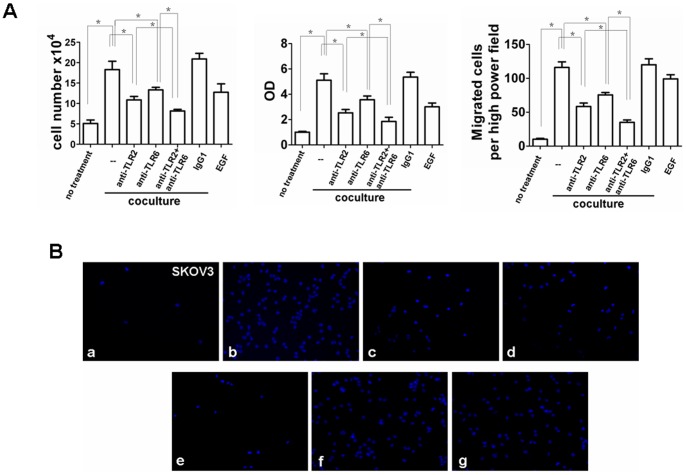
Inhibition of TLR2/6 decreases macrophage-induced ovarian tumor cell progression. Human peripheral blood monocyte-derived macrophages and SKOV3 cells were preincubated with 10 µg/ml anti-TLR2, 10 µg/ml anti-TLR6 or an IgG1 isotype control Ab (10 µg/ml) for 2 h. The cells were co-cultured in DMEM (10%HS) for 4 days. 100 ng/ml EGF was used as control. (A) Cell number, proliferation and invasion of SKOV3 cells were measured. Mean fold change ± SEM, n = 3, *p<0.05. (B) Matrigel invasion assay of SKOV3. DAPI staining of migrated tumor cells. Representative sections are shown as indicated. (a) SKOV3 no treatment. (b) SKOV3+macrophages. (c) SKOV3+macrophages+neutralizing anti-TLR2. (d) SKOV3+macrophages+neutralizing anti-TLR6. (e) SKOV3+macrophages+neutralizing anti-TLR2 and anti-TLR6. (f) SKOV3+macrophages+IgG1 isotype control Ab. (g) SKOV3+EGF (100 ng/ml).

We next sought to determine whether induction of hCAP18/LL-37 was dependent upon TLR2 induction. Human macrophages derived from peripheral blood monocytes were incubated for 2 h with a TLR2-neutralizing antibody or an IgG1 isotype control antibody, then stimulated for 24 h in SKOV3 cell conditioned media (SKOV3-CM) with 10% HS. In macrophage cells treated with the TLR2 neutralizing antibody a significant reduction in hCAP18/LL-37 induction was observed, no significant effect on gene induction was observed in the control antibody treated samples (Fig. 3A). To determine if induction of VDR, Cyp24, and Cyp27B1 was also influenced by exposure to the TLR2 neutralizing antibody, mRNA levels were measured. The samples exposed to the sameTLR2 antibody demonstrated no induction of VDR, Cyp24, or Cyp27B1, again no inhibitor affects were observed in the IgG antibody control samples (Fig. 3B-D). These experiments were repeated using a TLR6 neutralizing antibody, the same pattern of inhibition was observed, with induction of hCAP18/LL-37, VDR, Cyp24, and Cyp27B1 being blocked in the TLR6 antibody samples, but not the control group (Fig. 3A-D). Combining TLR2 and TLR6 neutralizing antibodies offered the possibility of a synergistic inactivation effect of these genes (Fig. 3A-D). These data indicate tumor-mediated induction of hCAP18/LL-37 requires TLR2/6 activity in macrophages. This induction mechanism is believed to be involved in vitamin D3 signaling. In further support of our model, the addition of neutralizing antibodies against TLR2 or TLR6 significantly suppressed SKOV3 cell proliferation and invasiveness (Fig. 4A, B). Combining TLR2 and TLR6 neutralizing antibodies resulted in a synergistic inhibition effect on SKOV3 cell progression (Fig. 4A, B). These results indicate activation of TLR2 or TLR6 is required for the tumor cell progression.

### TLR2/6 mediated expression of hCAP18/LL-37 via Cyp27B1 and VDR activation

**Figure 5:**
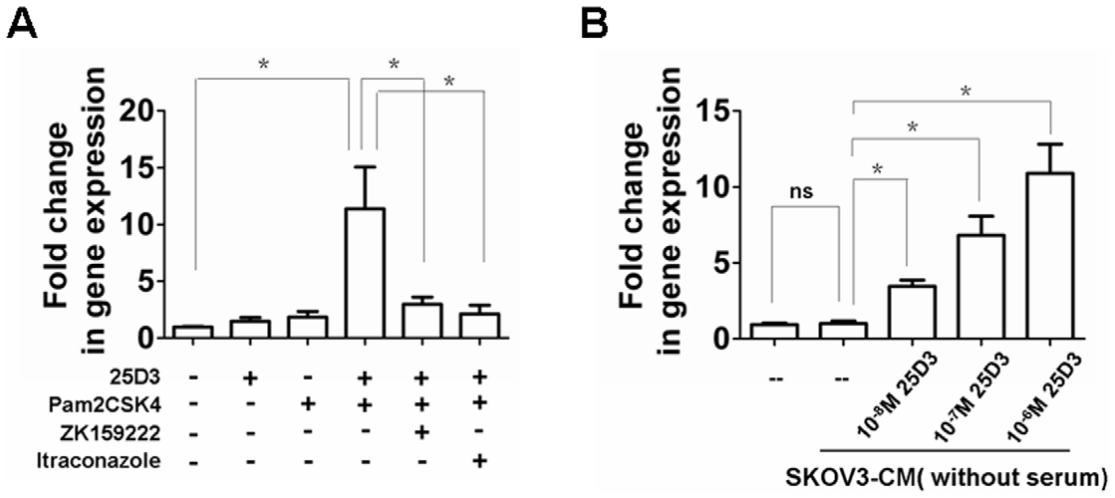
TLR2/6 ligand lead to an increase of hCAP18/LL-37 gene expression by activation of Cyp27B1 and VDR. (A)Human macrophages were pretreated with the VDR antagonist ZK159222 (10^−7^ M) or the Cyp27B1 antagonist itraconazole (10^−7^ M) for 2 h and then stimulated with TLR2/6 ligand Pam2CSK4 (100 ng/ml) in the presence or absence of 25D3 (10^−6^ M) (DMEM without serum) for 24 h. Expression of hCAP18/LL-37 mRNA was determined as described in Figure 3. (B) Regulation of hCAP18/LL-37 gene on stimulation with 25D3 in DMEM (without serum) or SKOV3-CM (without serum). Mean fold change ± SEM, n = 3, *p<0.05.

The Cyp27B1 dependent bioconversion of 25D3 to 1,25D3 is a key feature of vitamin D-mediated hCAP18/LL-37 expression [33]. To determine whether the tumor-induced activation of TLR2/6 enhanced bioactivity of Cyp27B1, thus leading to hCAP18/LL-37 expression, macrophages were exposed to the TLR2-TLR6 ligand Pam2CSK4, in the presence and absence of 25D3 media (DMEM without serum). Expression of hCAP18/LL-37 was not influenced when exposed to 25D3 or Pam2CSK4 independent of each other. However, hCAP18/LL-37expression was induced after simultaneous exposure (Fig. 5A). Moreover, inhibition of Cyp27B1 by itraconazole blocked TLR2/6 activation and a subsequent increase inhCAP18/LL-37 mRNA levels (Fig. 5A). Addition of the VDR antagonist ZK159222 also inhibited induction of hCAP18/LL-37 expression as determined by mRNA levels (Fig. 5A). Next, we co-cultured primary macrophages and SKOV3-CM (without serum) in presence of a range of 25D3 concentration, and levels of hCAP18/LL-37 mRNA were measured by qPCR. In another macrophage/SKOV3-CM co-culture experiment the addition of 25D3 significantly increased hCAP18/LL-37 mRNA levels in a dose dependent manner (Fig. 5B). It is noteworthy that SKOV3-CM without HS did not induce expression of hCAP18/LL-37 (Fig. 5B) because of insufficient vitamin D in culture media [24], [33]. These results therefore suggest that tumor-induced activation of TLR2/6 in human macrophages triggers hCAP18/LL-37 expression. Furthermore the data supports this up regulation is dependent on the Cyp27B1 and VDR mediated endogenous production of 1,25D3.

### Tumor-secreted versican V1 activates TLR2 and TLR6 to induce hCAP18/LL-37 expression in macrophages

**Figure 6:**
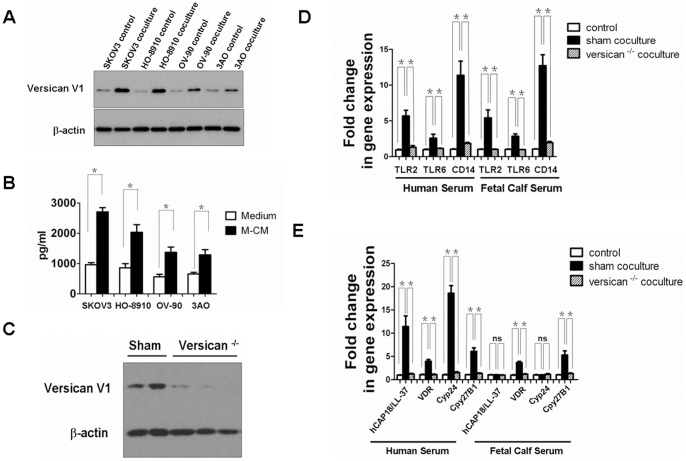
Tumor cell produced versican V1 induced hCAP18/LL-37 expression by activation of TLR2 signaling in macrophages. (A) Western blot of versican V1 protein in ovarian cancer cells after 24 h co-cultured with macrophages. (B) Versican V1 ELISA assay of cell supernatants from ovarian cancer cells with medium and after 24 h treatment with M-CM. (C) SKOV3 cells were transduced with versican V1 shRNA. After selection, expression of the indicated protein was analyzed. (D, E) Macrophages were co-cultured with sham shRNAs SKOV3 cells or versican V1 ^−/−^ SKOV3 cells for 24 h in medium with HS or FCS, and mRNAs expression levels of TLR2, TLR6, CD14 (D) and hCAP18/LL-37, VDR, Cyp24, Cyp27B1 (E) were measured.

As indicated above, co-culture of macrophages/SKOV3 cells activated TLR2/6 and thus increased hCAP18/LL-37 expression in macrophages. These results suggest undetermined soluble factors produced by the SKOV3 cells mediate this process. Kim et al. demonstrated that versican V1, a macrophage activator that acts through TLR2 and its coreceptors TLR6 and CD14, is up regulated in many human tumors including ovarian cancer and lung cancer, and enhances lung tumor metastatic growth [25]. To determine if versican V1 could be the soluble factor responsible for the TLR2/6 activation observed here we examined versican V1 expression levels in tumor cells co-cultured with macrophages. Figure 6A showed versican V1 protein expression was increased in total cell lysates of SKOV3, HO-8910, OV-90, and 3AO cells when co-cultured for 24 h. To investigate the level of versican V1 secretion from ovarian tumor cells we incubated SKOV3, HO-8910, OV-90, and 3AO cells with conditioned medium from macrophages (M-CM) for 24 h. ELISA assays were performed to determine the presence of secreted versican V1 in cell medium. After M-CM treatment, ovarian tumor cell secretion of versican V1 markedly increased (Fig. 6B). Versican V1 secretion was undetectable in M-CM by itself (data not shown).

**Figure 7:**
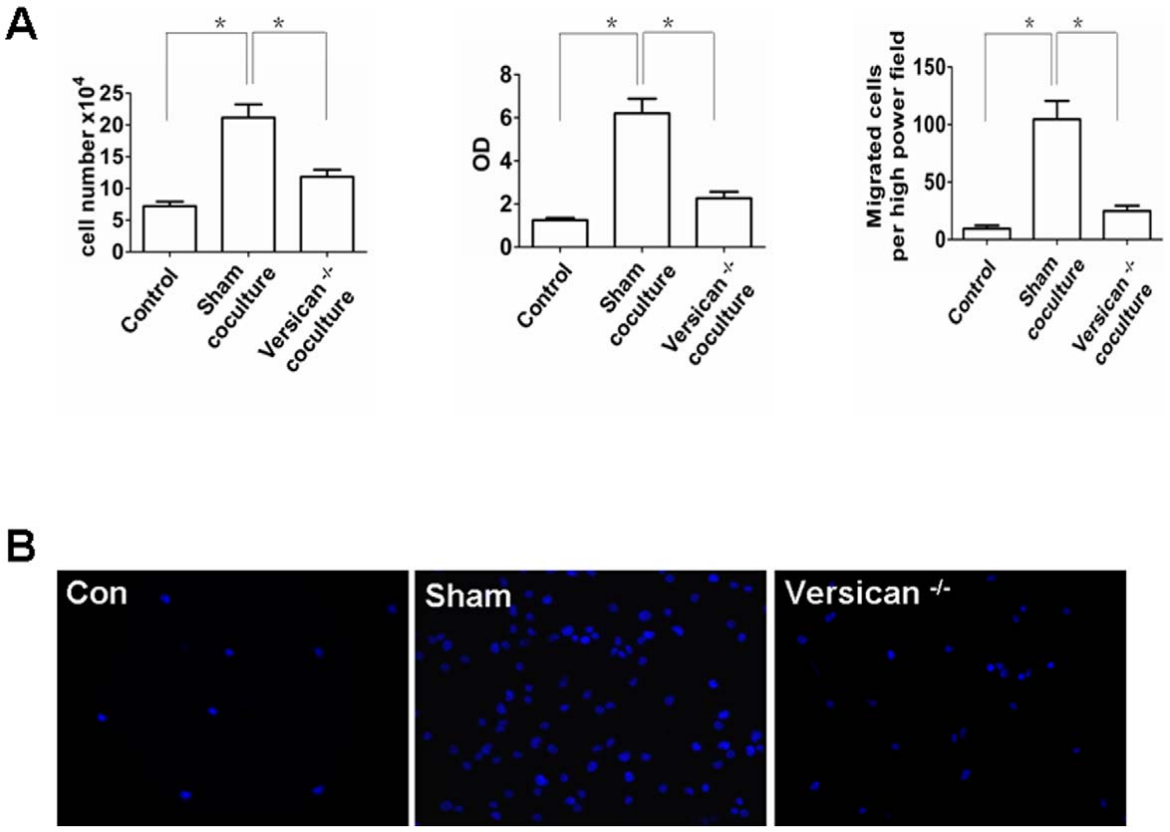
Knockdown of tumor cell versican V1 inhibits ovarian cancer cells proliferation and invasion. (A) Macrophages co-cultured with sham shRNAs SKOV3 cells or versican V1 ^−/−^ SKOV3 cells for 4 days, cell number, proliferation and invasion of SKOV3 cells were measured. Mean fold change ± SEM, n = 3, *p<0.05. (B) Matrigel invasion assay of SKOV3. DAPI staining of migrated tumor cells. Representative sections are shown as indicated. Con: SKOV3 control. Sham: sham shRNAs SKOV3+macrophages. versican V1 ^−/−^: versican V1 ^−/−^ SKOV3+macrophages.

We performed RNAi knockdown experiments to further assess versican V1 influence on macrophage TLR2/6 activation, hCAP18/LL-37 induction, and subsequent tumor cell proliferation and invasion. Stable SKOV3 cell lines expressing short hairpin RNA (shRNAs) specific for versican V1 were generated. Versican V1 protein knockdown was confirmed by Western blot (Fig. 6C). Macrophage cells were co-cultured with versican V1 ^−/−^ and sham shRNAs SKOV3 cell lines in HS medium. Induction of TLR2, TLR6, and CD14 as well as VDR, Cyp24, and Cyp27B1 was significantly reduced when macrophages were co-cultured with versican V1 ^−/−^ SKOV3 cell line (Fig. 6D, E). Furthermore, versican V1 knockdown resulted in reduction of hCAP18/LL-37 expression (Fig. 6E). To assess the role of vitamin D3 in versican V1 mediated induction of gene expression in macrophages co-culture experiments were conducted in medium with FCS (the 25D3 levels in FCS are one fifth of human serum). Here, versican V1 was capable of inducing TLR2, TLR6, CD14, VDR, and Cyp27B1, but not hCAP18/LL-37 and Cyp24. This data indicates vitamin D3 is essential for versican V1 mediated enhancement of hCAP18/LL-37 expression in macrophages. As expected, *in vitro* tumor cell proliferation and invasiveness was observed in versican V1 knockdown cells (Fig. 7A, B). These results strongly suggest TLR2/6 activation and subsequent upregulation of hCAP18/LL-37 are dependent on versican V1 expression in the tumor cells in this co-culture model.

## Discussion

Here we report the significance of versican V1 in enhanced hCAP18/LL-37 expression. The tumor cell secreted versican V1 influences hCAP18/LL-37 mediated *in vitro* tumor promotion and cell invasion through TLR2/6 activation and vitamin D-dependent mechanisms in macrophages.

A strong association between inflammation and cancer is supported by an ever expanding body of clinical and experimental observations [2], [3]. Inflammation is hypothesized to cause DNA alterations and induces oncogenic mutations via production of reactive oxygen and nitrogen species. From these mutations tumor initiation arises [1], [34]. Furthermore, inflammation promotes tumor progression via cytokines produced by infiltrating immune cells, such as macrophages and neutrophils [1]. The interactions between neoplastic cells and their microenvironment, include macrophages, is crucial in each stage of tumorigenesis [1], [35]. There is considerable evidence that macrophages are essential for tumor growth, invasion and metastasis of ovarian and lung cancer among others [29], [34], [36]. Given these findings it is not surprising that macrophages have been linked to enhanced growth and invasiveness in ovarian cancer cells. However, the underlying molecular mechanisms of this process are defined. Here we examined the role macrophages play in stimulating ovarian cancer cells growth and invasiveness using an *in vitro* assay. Our data strongly suggests the human cationic antimicrobial protein hCAP18/LL-37 has a critical function in macrophage mediated cancer progression.

Previous reports showed that hCAP18/LL-37 expression is up regulated in ovarian tumors and promotes tumor progression through direct stimulation of tumor cells, initiation of angiogenesis and recruitment of immune cells [14], [18], [19]. Our current research supports these finding, in that a significant increase in macrophage hCAP18/LL-37 induction, when co-cultured with ovarian tumor cell SKOV3. This increased expression of hCAP18/LL-37 is exclusive to macrophages in this body of research, no induction of SKOV3 hCAP18/LL-37 was detecting in any of these experiments. Thus macrophages, not cancer cells, contribute the enhanced release of hCAP18/LL-37 into tumors' microenvironments. This increase in total hCAP18/LL-37 levels may then influence tumor cell progression.

Although the biologic role of LL-37 in tumor-promotion has been partly elucidated, to our knowledge, the elements responsible for control of expression within tumor microenvironments were not previously reported. A large portion of inflammatory mediators, including TNFα, IL-6, IL-8, and INF-γ, do not induce expression of hCAP18/LL-37 [23]. Some cytokines and growth factors, such as LPS, IL-1αα and insulin-like growth factor-1 in keratinocytes, however, have all been reported to induce hCAP18/LL-37 expression [37], [38]. Gombart et al. reported that VDR mediates a strong up-regulation of hCAP18/LL-37 in response to 1,25D3 or its analogs in human macrophages [23]. In addition, TLR2/1 activation of human macrophages is known to induce expression of VDR and Cyp27B1 and is required for downstream hCAP18/LL-37 production [24]. These observations led us to examine involvement of vitamin D3 in macrophage expression of hCAP18/LL-37. Research by Liu et al. showed that 25-hydroxyvitamin D3 (25D3, the 1,25D3 precursor) levels in human serum (HS) were five times higher than those in fetal calf serum (FCS). Given the predicted role of vitamin D3 in hCAP18/LL-37 expression these findings suggests human cells cultured in HS may be critical for study of hCAP18/LL-37 function and expression *in vitro* [24]. Therefore, we used HS in the majority cell culture experiments for this study. We found up-regulation of VDR and VDR-related genes, including Cyp24, Cyp27B1, in macrophages when they were co-cultured with SKOV3 cells. These VDR related genes are involved in vitamin D3 production and regulation. Cellular levels of 1,25D3 are controlled by synthesis via CYP27B1 and degradation via CYP24. CYP24 gene was previously shown to be inducible by 1,25D3 in macrophages [24], [33]. In addition, the expression of TLR2 and its coreceptors TLR6 and CD14, also increased in macrophages. Furthermore, our results suggested that induction of VDR, Cyp24, Cyp27B1 and hCAP18/LL-37 requires macrophageTLR2/6 activity in tumor microenvironments.

Previously, it has been reported that TLR2/1-regulated extra-renal utilization of 25D3 in macrophage is required for hCAP18/LL-37 expression [32]. We found that TLR2/6 activation in macrophages triggered induction of the Cyp27B1. This activation also enhanced bioconversion of 25D3 to 1,25D3 and in turn induces the expression of hCAP18/LL-37. We propose this to be a key step in the up regulation of hCAP18/LL-37, given that a specific Cyp27B1 inhibitor blocked the TLR2/6-mediated induction of hCAP18/LL-37. We found hCAP18/LL-37 over-expression in macrophages requires specific elements of the tumor micromilieu, such as vitamin D3 precursors not typically present in cell culture system. Without inclusion of 25D3, the function of Cyp27B1 would not have been detected, and TLR2/6 would not have influenced the induction of hCAP18/LL-37 in this setting.

In ovarian cancers, versicanV1, an aggregating chondroitin sulphate proteoglycan, has been shown to accumulate both in cancer cells and tumor stroma. Elevated versican levels have been observed in primary ovarian carcinoma tumors and secondary metastases when compared with normal ovaries [39], [40]. It has been identified as a tumor-derived factor capable of causing decreased cell/cell and cell/matrix adhesion, thus facilitating tumor invasion and metastasis [25], [40]. Furthermore, a recent study demonstrated that versican V1 was a macrophage activator. Specifically acting through activation of TLR2 and its coreceptors TLR6 and CD14, and furthering to lung tumor metastatic growth [25]. Data presented here demonstrates ovarian tumor cells up-regulated versican V1 expression when co-cultured with macrophages. This up-regulation is believed to be the primary mechanism that stimulates activation of TLR2/6 in macrophages. Blockage of versican V1 by shRNA knockdown, in tumor cells, inhibited TLR2/6, VDR, Cyp24, Cyp27B1, and hCAP18/LL-37 induction, as well as tumor cell proliferation and invasiveness. Furthermore, these results supported the hypothesis that Vitamin D3 is essential for versican V1 to induce expression of hCAP18/LL-37 in macrophages. Interestingly, versican V1 induced expression of TLR2, TLR6, CD14, VDR, and Cyp27B1 is independent on Vitamin D3. Our results outline a novel mechanism for hCAP18/LL-37 expression in macrophages is regulated via versican V1 secretion from tumor cells into the microenvironment.

**Figure 8:**
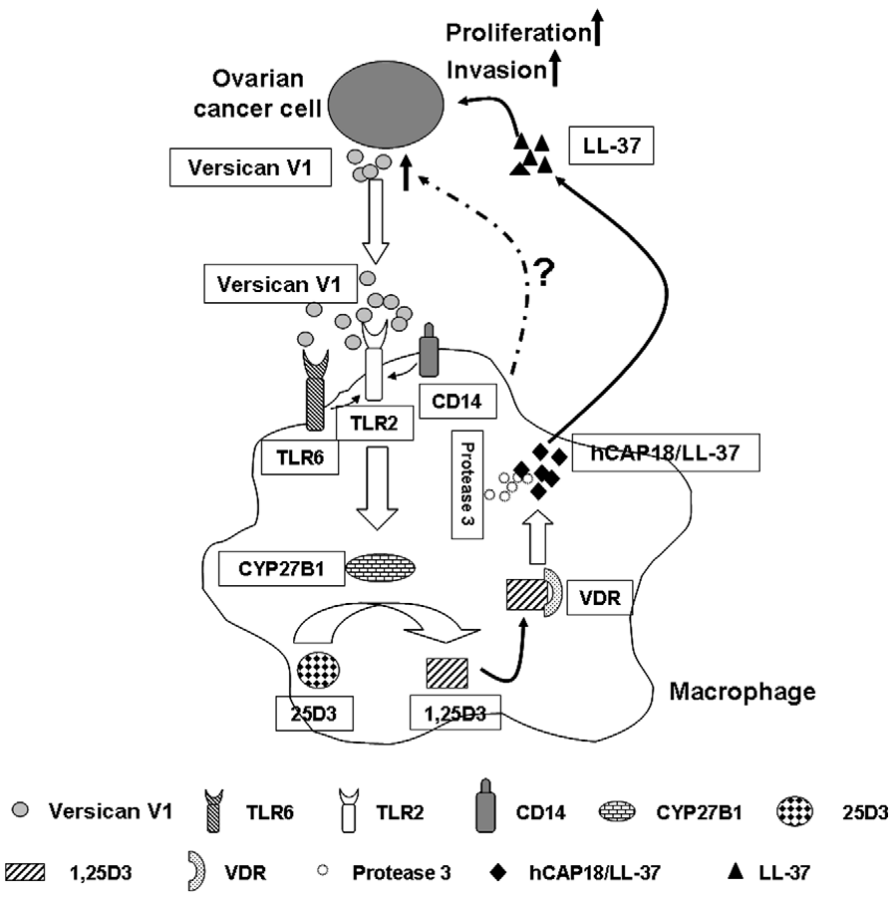
Proposed model of hCAP18/LL-37 up regulation as mediated by tumor-macrophage interactions. In tumor microenvironment, macrophages are activated by versican V1 produced by cancer cells, which then leads to activation of TLR2/6 and subsequent induction of Cyp27B1. As a consequence 25D3 is converted to 1,25D3, which then activates VDR, resulting in increased macrophage hCAP18/LL-37 expression. The released LL-37 initiates a tumor-promoting effect and increases tumor proliferation and invasion.

In summary, the results presented here suggest a novel mechanism for hCAP18/LL-37 expression involving tumor-macrophage interaction. As shown in Figure 8, versican V1 produced by ovarian tumor cell leads to activation of TLR2 and its coreceptors TLR6 and CD14 in macrophages. The increased TLR2 signaling increases Cyp27B1 induction and subsequently induces the conversion of inactive 25D3 into the active 1,25D3. The serum level of 25D3 in human are approximately 7.5×10^−8^ M and sufficient for the induction of hCAP18/LL-37 in macrophages [24], [32]. Thus, hCAP18/LL-37 generated by 1,25D3 in tumor microenvironments propagates a tumor-promoting effect increases tumor proliferation and invasion. This model of tumor-promoting effects and the underlying mechanisms responsible for hCAP18/LL-37 up regulation may provide potential points for anti-tumor intervention.
